# Principal Component Analysis Applied to Radiomics Data: Added Value for Separating Benign from Malignant Solitary Pulmonary Nodules

**DOI:** 10.3390/jcm12247731

**Published:** 2023-12-17

**Authors:** Birte Bomhals, Lara Cossement, Alex Maes, Mike Sathekge, Kgomotso M. G. Mokoala, Chabi Sathekge, Katrien Ghysen, Christophe Van de Wiele

**Affiliations:** 1 Department of Diagnostic Sciences, University Ghent, 9000 Ghent, Belgium; birte.bomhals@ugent.be (B.B.); lara.cossement@ugent.be (L.C.); 2 Department of Morphology and Functional Imaging, University Hospital Leuven, 3000 Leuven, Belgium; alex.maes@azgroeninge.be; 3 Department of Nuclear Medicine, Katholieke University Leuven, AZ Groeninge, President Kennedylaan 4, 8500 Kortrijk, Belgium; 4 Department of Nuclear Medicine, Steve Biko Academic Hospital and Nuclear Medicine Research Infrastructure (NuMeRi), University of Pretoria, Pretoria 0002, South Africa; 5 Department of Pneumology, AZ Groeninge, 8500 Kortrijk, Belgium

**Keywords:** solitary pulmonary nodules, texture features, principal component analysis

## Abstract

Here, we report on the added value of principal component analysis applied to a dataset of texture features derived from 39 solitary pulmonary lung nodule (SPN) lesions for the purpose of differentiating benign from malignant lesions, as compared to the use of SUVmax alone. Texture features were derived using the LIFEx software. The eight best-performing first-, second-, and higher-order features for separating benign from malignant nodules, in addition to SUVmax (MaximumGreyLevelSUVbwIBSI184IY), were included for PCA. Two principal components (PCs) were retained, of which the contributions to the total variance were, respectively, 87.6% and 10.8%. When included in a logistic binomial regression analysis, including age and gender as covariates, both PCs proved to be significant predictors for the underlying benign or malignant character of the lesions under study (*p* = 0.009 for the first PC and 0.020 for the second PC). As opposed to SUVmax alone, which allowed for the accurate classification of 69% of the lesions, the regression model including both PCs allowed for the accurate classification of 77% of the lesions. PCs derived from PCA applied on selected texture features may allow for more accurate characterization of SPN when compared to SUVmax alone.

## 1. Introduction

A solitary pulmonary nodule (SPN) is defined as a well-marginated parenchymal lesion less than 3 cm in diameter that is completely surrounded by pulmonary parenchyma in the absence of any other lung abnormality [1]. Reported estimates of the prevalence of SPN identified on chest computed tomography vary from 2% to 69% [2]. Differential diagnosis of SPNs includes both benign etiologies, e.g., hamartoma, tuberculosis, and infection, as well as primary lung cancer and distant metastasis [3,4]. Reported malignancy prevalence rates in SPNs have varied from 7% to 40% [2]. Given that the early detection of lung malignancy is of paramount importance, non-invasive techniques allowing for the accurate differentiation of benign from malignant solitary lung nodules are of major clinical interest. 18F-FDG (fluoro-deoxyglucose) PET (positron emission tomography)/CT imaging is currently widely used to characterize SPN, with a standardized uptake value greater than 2.5 g/mL being indicative of malignancy [5]. However, its diagnostic efficacy does not fully meet clinical needs, and its specificity in granuloma-endemic regions is much lower than that in nonendemic regions [6].

In recent years, radiomics, the process of extracting and analyzing textural features from medical images, including 18F-fluorodeoxyglucose (FDG)/PET CT imaging, has gained wide clinical interest, and some of the features studied aside from SUVmax have been shown to hold promise for the characterization of SPNs [7,8,9,10]. The introduction of radiomics in nuclear medicine imaging has been made possible via several developments in image processing and analysis, such as denoising and correction of partial volume effects and (semi)automated lesion delineation methods. Importantly, many of the extracted features have proven to be highly correlated, especially when studying low-volume lesions, as is the case for SPNs [11,12]. The inclusion of highly correlated texture features in a regression model may lead to instability of the regression coefficients weights with small changes in the data leading to very different regression coefficients, a phenomenon known as “the bouncing betas” [13]. Thus, the generated model results will be unstable, vary a lot with small changes introduced in the dataset, and may drop significantly in accuracy when applied to another sample of data.

Principal component analysis (PCA) is a technique for reducing the dimensionality of large datasets containing highly correlated variables. It does so by creating new uncorrelated variables that successively maximize variance, minimize information loss, and avoid the bouncing beta phenomenon [14]. Here, we report on the added value of PCA applied to a dataset of texture features derived from SPN lesions for the purpose of differentiating benign from malignant SPN when compared to the use of SUVmax alone.

## 2. Patients and Methods

### 2.1. Patients

This retrospective study was approved by the Ethics Committee of our Hospital (AZGS2022052), and informed consent was obtained prior to inclusion. Thirty-nine patients presenting with a solitary pulmonary nodule referred for 18F-FDG PET/CT imaging between January and December 2020 were included in the study. There were 20 men and 19 women (mean age 39 years, range: 25–92 years). The final diagnosis was established using biopsy and surgical removal and long-term follow-up of at least 24 months (range 24–37 months).

### 2.2. Data Acquisition, Reconstruction, and Tumor Segmentation

A whole-body FDG PET/CT scan using a GE 64 mCT scanner was performed in all patients following a fasting period of at least 8 h prior to imaging, thus ensuring a serum glucose level of less than 200 mmol/L. The injected dose was 7 MBq/kg body weight, and the time interval between injection and acquisition was 60 ± 7 min. PET raw data were acquired for 1 min per bed position from the top of the skull to the proximal third of the femora. For CT imaging, a tube voltage of 120 kV and a tube current ranging from 80 to 180 mAs were applied (automatic setting). PET image reconstruction was performed using time of flight (TOF) and ordered subset expectation maximization (OSEM). Point spread function (PSF) correction was performed using the QCLEAR software. The image matrix used was a 128 × 128 matrix. 18F-FDG uptake was estimated using decay correction normalized to injected dose and patient body weight, yielding customary standardized uptake values (SUVs)

Region growing and a fixed threshold set to 30% of the SUVmax in the lung lesions were applied on the OSEM + PSF + TOF generated images in order to obtain volumes of interest. The latter images were chosen given that they produce better image quality in terms of the signal-to-noise ratio, contrast, and lesion detectability. The minimal lesion volume included for subsequent analysis was 4 cm^3^. Some texture parameters are based on a series of neighboring voxel values in the x, y, or z directions. Accordingly, a series of less than 4 voxels (minimal volume of at least 4 × 4 × 4 or 64 voxels) corresponding to a minimal volume required of at least 4 cm^3^ with the matrix chosen would not make the calculations meaningful.

### 2.3. Texture Analysis

First-, second- -and higher-order features were obtained using the readily accessible and easy-to-use LIFEx software (www.lifexsoft.org (accessed on 2 October 2022)) [15]. Second- and higher-order features were calculated using the gray level co-occurrence matrix (GLCM), the gray-level run length matrix (GLRLM), the gray-level size zone matrix (GLSZM), and the neighboring gray-tone difference matrix (NGTDM). Original PET values were resampled to 64 gray levels or bins in order to reduce image noise. A quantization of 64 gray levels was previously shown to provide the best compromise between the sufficient sampling of voxel SUVs, preservation of original intensity information, and potential complementary information with respect to the metabolic active lesion volume. In total, 118 features were obtained from the images.

### 2.4. Statistical Analysis

#### Statistical Analysis Was Performed Using SPSS Version 28

Prior to analysis, all texture features were normalized (texture feature result—texture feature mean/texture feature standard deviation). Thus, normalized texture feature data were used for principal component analysis (PCA). Given the high number of features obtained using the LIFEx software when compared to the number of patients included, ROC curve analysis was performed to define the normalized features that allowed for more optimal separation of benign from malignant lesions for first-, second-, and higher-order features. For the purpose of conducting a principal component analysis, various recommendations regarding the appropriate sample size to use have been forwarded, with the minimum number of subjects per variable advocated ranging from 2 to 5 subjects per variable [16,17]. Thus, taking into consideration the sample size studied, out of the 118 features generated by the LIFEx software, we selected the 8 best-performing first-, second-, and higher-order features, also taking into consideration the various matrices, based on the AUC values derived from ROC analysis for separating benign from malignant nodules for inclusion in the PCA, in addition to SUVmax (MaximumGreyLevelSUVbwIBSI184IY). These features were subsequently used for PCA analysis (see Table 1).

To assess the suitability of the dataset for factor analysis (a value > 0.6 was deemed significant), the Kaiser–Meyer–Olkin (KMO) test was used. To assess whether the correlation matrix of the normalized texture features proved significantly different from an identity matrix in which correlations between variables were all zero (a *p*-value < 0.05 was deemed significant), we applied Bartlett’s test. Varimax rotation was used to maximize the sum of square loadings.

The squared multiple correlations between the newly generated principal components and all other texture features, also termed commonalities, were considered significant when higher than or equal to 0.5 or lower than or equal to −0.5. Based on the commonalities, original texture features that were highly correlated with derived PCAs were then identified.

Binomial logistic regression was subsequently performed, including gender and age as covariates, to assess whether the principal components identified were independent predictors of the underlying character of the lesions and, if so, whether the regression model was better performing than SUVmax alone in separating benign from malignant lesions.

## 3. Results

In total, 20 lesions were benign (3 granulomas, 2 non-specific inflammations, 1 fibrosis, 10 reduced nodules, and 4 stable nodules), and 19 proved malignant (10 adenocarcinomas, 1 large cell carcinoma, 6 non-small cell lung carcinomas, and 2 squamous cell carcinomas).

The following texture features (IBSI) were selected based on their AUC values, taking into consideration the various matrices studied: integrated intensity (IBSI99NO, 0.787), intensity-based maximum gray level or SUVmax (IBSI84IY, 0.808), GCLM joint average (IBSI60VM, 0.839), GCLM autocorrelation (IBSIQWB0, 0.839), GLRLM High Gray Level Run Emphasis (IBSIG3QZ, 0.839), GLRLM Short Run High Gray Level Emphasis (IBSIGD3A, 0.839), NGTDM Contrast (IBSI65HE, 0.837), NGTDM Complexity (IBSIHDEZ, 0.811), and GLSZM High Gray Level Zone Emphasis (IBSI5GN9, 0.803). Their normalized equivalents were included in the PCA. All non-normalized features proved significantly higher in malignant when compared to benign lesions (*p*-values ranging from 0.001 to 0.002).

The KMO measure of adequacy was 0.756, and Bartlett’s test yielded a *p*-value < 0.001, allowing for principal component analysis.

Two principal components (PCs) were retained: one PC with an eigenvalue of 7.887, which proved highly correlated with eight of the included normalized features but especially with contrast and complexity from the NGTDM matrix, and one factor with an eigenvalue of 1, which proved highly correlated with morphological integrated intensity (IBSI99N0, r = 0.976) (see the rotated component matrix in Table 1).

The contribution to the total variance of these two principal components derived using Varimax rotation was, respectively, 87.6% and 10.8%. The two principal components together thus explained 98.4% of the total variance (cumulative variance).

When including both PCs in a logistic binomial regression analysis, including age and gender as covariates, both PCs were retained as significant (*p* = 0.009 for the first PC and 0.020 for the second PC) (see Table 2). As opposed to SUVmax alone (MaximumGreyLevelSUVbwIBSI184IY), which allowed for the accurate classification of 69% of the lesions, the regression model including both PCs allowed for the accurate classification of 77% of the lesions (see Table 3).

## 4. Discussion

Using principal component analysis, the dataset of nine selected texture features generated by the LIFEx software could be compressed to a dataset of two new uncorrelated variables or principal components while maintaining 98.4% of the total variance. The first principal component, accounting for 87.6% of the total variance, proved to be highly positively correlated with eight of the nine features included for PCA; however, the highest correlation was found between NGTDMcontrast (IBSI165HE) and NGTDM complexity (IBISHDEZ). Features derived from the NGTDM, such as contrast and complexity, have been previously shown to be fundamental parameters of image textures known to correlate with human perception of texture within an image [18]. NGTDM features aim at quantifying the sum of differences between the gray level of a pixel of a voxel and the mean gray level of its neighboring pixels or voxels within a predefined distance. Contrast is a measure of the spatial intensity change but is also dependent on the overall gray level dynamic range. Contrast is high when both the dynamic range and the spatial change rate are high, i.e., an image with a large range of gray levels (SUV levels), with large changes between voxels and their neighborhood. Related to this, complexity describes how common the non-uniform and rapid changes in Graylevels are. In our study, both contrast and complexity derived from the NGTDM proved to be significantly higher in malignant as opposed to benign lesions. FDG was previously shown to accumulate in activated inflammatory cells that are dispersed over the site of inflammation/infection, with neutrophils being the cell responsible for the larger part of the FDG PET signal in both acute and chronic inflammatory responses in the lung [19]. The more densely clustered aspect of tumor cells when compared to inflammatory cells may, in part, explain the higher contrast in malignant lesions as identified in our study. Our findings highlighting the importance of features derived from the NGTDM matrix for the characterization of solitary lung nodules are in line with a previous study by Chen et al., who, similar to us, found that adding information from the NGTDM to SUVmax increased the discriminatory power of FDG PET imaging to separate benign from malignant solitary lesions [20]. In their study, the authors first visually scored all lung lesions on the FDG PET/CT examination using a 5-point scale, including information derived from both FDG PET and CT imaging and subsequently after information from the NGTDM features was provided. Of interest, textural features derived from the NGTDM matrix have been previously shown to allow for the differentiation of primary and nodal tumors from normal tissue in head and neck cancer, to allow for delineation of radiotherapy plans, and to predict response and outcome to treatment in colorectal cancer as well as non-small cell lung carcinoma [21,22,23,24]. Yu et al. developed a co-registered multimodality pattern analysis segmentation system to automatically delineate radiation targets in head and neck cancer. The inclusion of coarseness and busyness derived from FDG PET images in a decision-tree-based K-nearest-neighbor classifier allowed for a more accurate and consistent delineation of both primary tumor tissue and involved lymph nodes when compared to threshold-based methods [21]. In a study by Oh et al., including patients suffering from hypopharyngeal squamous cell carcinoma, responders to radiochemotherapy showed a lower coarseness and busyness when compared to non-responders [22]. Lovinfosse et al. performed texture analysis on FDG PET images obtained prior to neoadjuvant treatment in eighty-six patients suffering from locally advanced rectal cancer. Whereas the texture feature coarseness was significantly associated with disease-free survival, the feature’s dissimilarity and contrast proved significantly associated with overall survival in the multivariate analysis [23]. Finally, in the series by Cook et al., including fifty-three patients suffering from non-small cell lung carcinoma who were treated with radiochemotherapy and had a pretreatment FDG-PET examination performed, the texture feature coarseness proved an independent predictor of overall survival in multivariate analysis, whereas contrast and busyness were associated with progression-free survival [24]. Of interest, the correlation found between the first PC and SUVmax is also not surprising, given the well-established clinical utility of SUVmax to differentiate benign from malignant nodules. FDG uptake was previously shown to be proportional to the glucose utilization rate, with malignant tissue consuming significantly higher levels of glucose based on the Warburg principle when compared to normal tissue [25]. Furthermore, normal and inflammatory tissue exhibit faster glucose clearance based on their relatively higher levels of hexokinase relative to glucose-6-phosphatase, and malignant lesions continue to accumulate more FDG over time when compared to normal tissues [26].

The second PC proved very highly correlated with morphological Integrated intensity or metabolic tumor volume. In this series, as in others addressing the added value of radiomics in FDG PET/CT imaging, volumes smaller than 5 cm^3^ were not included for analysis. First, the qClear software used in this study overestimates SUV-max values for lesions below 22 mm in diameter (or a corresponding volume of 5 cm^3^). It thus underestimates the tumor volume when using region growing [27]. Second, some texture features require a series of at least 4 neighboring voxel values in the x, y, or z directions or a volume of at least 64 voxels. When using a voxel size of 4 mm and assuming sphere-like lesions, this corresponds to a volume of 4 cm^3^ or a lesion with a radius of 1 cm or a diameter of 2 cm [12,28]. Thus, in this study, as in others using radiomics for analysis of solitary pulmonary nodules, only lesions between 2 and 3 cm in diameter were included, and the results obtained only apply to those lesion sizes under study. Given the narrow range of diameter size, the high correlation found between the second PC and MTV suggests that the SUV mean is probably also of clinical significance in this specific setting. MTV was previously shown to bear both predictive and prognostic value in a wide variety of solid tumors. The limited range of lesion diameters under study may be considered a drawback; as shown by Khalaf et al., small benign pulmonary lung nodules (<1 cm) tend to have comparable SUV max values to malignant nodules with an accuracy of and SUVmax value of 2.5 of only 54% [29].

Importantly, when including both PCs in a logistic regression analysis, both proved to be significant predictors of the underlying character of the lesions (benign versus malignant). Given that both PCs are, by definition, orthogonal, they avoid the problem of collinearity. The possibility of reducing the nine features included to a new dataset of two new uncorrelated variables while maintaining the bulk of the variance is of major relevance for regression analysis, requiring a minimum number of 10 to 15 patients per predictor to produce reasonably stable estimates [30,31], which in our series would have left room for the inclusion of three variables at best. Importantly, the inclusion of the two PCs in the regression model led to an improved diagnostic accuracy as compared to the use of SUVmax values alone, with a gain of 8% in classification accuracy, in line with the findings by Chen et al. [20].

## 5. Shortcomings

In this study, we used a 30% fixed threshold region growing method for tumor delineation. It cannot be excluded that different results may be obtained when using a gradient-based method. Unfortunately, software algorithms allowing for gradient-based tumor delineation are not widely available, and currently, their use is mainly limited to those research centers where they were developed [32]. As pointed out previously, the results obtained in this series only apply to lesions with a diameter ranging from 2 to 3 cm, thus omitting a large number of lesions from analysis in whom differentiation based on SUVmax values is suboptimal. Also, radiomics models require separate training and test datasets. However, due to the limited sample size in this study, all available data were utilized for training purposes. Consequently, there is a lack of dedicated test data to evaluate the model’s performance. This could potentially lead to an overestimation of the model’s capabilities due to overfitting. Finally, this study is a retrospective one including a limited number of patients, and confirmation of our findings in larger patient populations is mandatory.

## 6. Conclusions

In this study on a series of 39 solitary pulmonary nodules with a range in lesion size varying from 2 to 3 cm, applying PCA reduced the dataset of nine selected texture features derived using the LIFEx software to a set of two uncorrelated new variables, while maintaining 98.4% of the total variance contained within the dataset. These two new uncorrelated variables were either highly correlated to features derived from the NGTDM matrix (first PC) or MTV (second PC) and proved to be independent predictors for characterization of the underlying nature (benign or malignant) of the nodules under study using logistic regression. The logistic regression model obtained using both PCs allowed for a more accurate classification of lung nodules as opposed to the use of SUVmax alone.

## Figures and Tables

**Table 1:** Rotated component matrix (correlations between the principal components (PC) and the included features; only *p*-values > 0.5 and <−0.5 are reported).

	PC1	PC2
Morphological integrated intensity	-	0.976
Intensity-based maximum gray level (SUVmax)	0.838	0.513
GCLM joint average	0.845	0.513
GCLM autocorrelation	0.830	0.552
GLRLM High Gray Level Run Emphasis	0.826	0.559
GLRLM Short Run High Gray Level Emphasis	0.830	0.553
NGTDM Contrast	0.981	-
NGTDM Complexity	0.927	-
GLSZM High Gray Level Zone Emphasis	0.816	0.575

**Table 2:** Results of the regression analysis, including the principal components.

	B	S.E.	Wald	Sig.
REGR factor score 1	2.532	0.964	6.899	0.009
REGR factor score 2	6.328	2.713	5.442	0.020
Constant	3.022	1.305	5.363	0.021

**Table 3:** (a) Classification based on intensity-based maximum gray level (SUVmax) alone. (b) Classification based on PC1 and PC2.

(**a**)			
	**Benign Predicted**	**Malignant Predicted**	**Percentage Correct**
Benign observed	16	4	80.0
Malignant observed	8	11	57.9
Overall percentage			69.0
(**b**)			
	**Benign Predicted**	**Malignant Predicted**	**Percentage Correct**
Benign observed	17	3	85.0
Malignant observed	6	13	68.4
Overall percentage			76.9

## Data Availability

Data may be obtained via the last author following a reasonable request and following approval from our Ethics Committee.
